# Visual Expressions of Children’s Strengths, Difficulties and Wishes in Person Picking an Apple from a Tree Drawings among Preschoolers Living in Areas of Persistent Political Violence

**DOI:** 10.3390/children9091387

**Published:** 2022-09-14

**Authors:** Michal Bat Or, Rafi Ishai, Nirit Barkay, Or Shalev

**Affiliations:** 1 School of Creative Arts Therapies, Emili Sagol Creative Arts Therapies Research Center, University of Haifa, Haifa 3498838, Israel; 2 Department of Psychology, The Program of Psychoanalytic Psychotherapy, Haifa 3498838, Israel; 3 Junior-High and High School Meshutaf Hof Hacarmel Yahad, Maagan Michael 3780500, Israel; 4 School of Creative Arts Therapies, University of Haifa, Haifa 3498838, Israel

**Keywords:** PPAT, children, self-potency, EF, maternal distress, political violence

## Abstract

The present study sought to inquire into the subjective experience of 156 preschoolers (age 4–6.9 years) living in an area of political violence in Israel (on the border with the Gaza Strip) during a period of massive bombing. Children were invited to draw a Person Picking an Apple from a Tree (PPAT), and were interviewed on their sense of self-potency using the CAMP, a measure of potency. Teachers were asked to report problems in executive functions using a few BRIEF scales; and mothers filled out a questionnaire for maternal distress (BSI), a measure of their child strengths and difficulties (SDQ), and were asked to provide their assessment regarding the extent to which their child was exposed to political violence. Findings reveal associations between mothers’ distress, the degree of exposure of their child to trauma, and the child’s emotional symptoms. PPAT analysis identified four main factors: Tree Generosity, Person Agency, Vividness, and As-Real-R. Positive associations were found between self-potency and the main factors of the drawings; negative associations were found between the child’s difficulties in executive functions and the drawing’s four main factors; and two small negative associations were found between the child’s emotional symptoms and Tree Generosity and As-Real-R factors. The following associations were found within each gender group: mothers’ depression degree was associated with boy’s Tree Generosity, and mother’s perceptions of their girl’s exposure to trauma was related to Person Agency, Tree Generosity, and As-Real-R factors; furthermore, a significant difference was found between the narrative focus of drawings in this sample and the narrative focus of drawings of a sample of the same age group from a non-war zone. In addition, narrative focus was found to be related to children’s self-potency. The discussion deals with the study’s findings through the prism of developmental psychology, self-agency, object-relations, and art-therapy theories.

## 1. Introduction

### 1.1. Civilians Living in Conditions of Persistent Political Violence

Civilians living in conflict-affected settings, who experience war, terror, military attacks and more, are exposed, as individuals and as a community, to immediate and unforeseen threats and danger. These situations were found to be associated with civilians’ reduced sense of safety, heightened anxiety, and post-traumatic stress symptoms—PTSD (e.g., [1,2,3]). PTSD, according to the DSM-V, appears after an exposure to traumatic event/s, and symptoms include re-experiencing, avoidance, negative mood and cognitions, and hyperarousal [4]. Since ongoing political violence causes an accumulation of stressful events over time, its impact can be explained by the Allostatic Load Framework that shows that the more people are exposed to stressful or traumatic events, the more they are at risk [5]. Diamond et al. [6] described the phenomenon of Ongoing Traumatic Stress Response (OTSR) as a unique outcome for some people who live for years in conditions of political violence. OTSR is different from PTSD in four aspects: (a) clients describe their anxiety symptoms as the cumulative result of repeated ongoing stressful events, and not as related to a marked traumatic event; (b) fewer re-experiencing symptoms appear; instead, the fear is focused more on daily life routines such as those that require leaving the house (for example- picking up the children); (c) Client’s patterns of fear and avoidance are reality-based, and thus can be considered reasonable; and (d) clients report a marked decrease of symptoms when they are away from the war zone. 

The present study focused on preschool children who were born into a situation of persistent political violence that began in the area where they reside about 12 years before they were born. It may be thus assumed that their parents, who were about to serve as a protective envelope to their children, experienced their safety as compromised, due to constant uncertainty and alertness. It is well known that young children’s development, health, and well-being depend on their caregivers; for example, research showed that the mother’s well-being serves as a resilience factor [7,8,9], while maternal stress was associated with children’s internalizing and externalizing disorders [10]. In a systematic review on the effects of political violence on young children [11], parental care was found as a moderator of the associations between exposure to violence and children’s outcomes. For this reason, we included in the current study a measure of the degree of maternal stress, as this maternal aspect may have a significant impact on the child. Since the missile attacks occur suddenly in the child’s daily life, and are uncontrollable, there is a high risk that these children’s development and resiliency will be impacted by the trauma in these living conditions (e.g., [12,13]).

### 1.2. Children’s Emotional-Behavioral Aspects in Political Violence Settings

Since events of political violence mainly function as threats and stressors in the children’s daily life, they can elicit fear and anxiety and other overwhelming feelings that arouse a sense of helplessness. These emotional states may be seen as a normal reaction to an abnormal situation [12]. However, prolonged exposure to political violence in childhood may limit children’s adaptation [14]. Research shows that children who live in an environment of political violence tend to suffer more from clinical behavioral and emotional distress [15,16,17]; PTSD; psychosomatic symptoms; sleep disorders; and disturbed play [11]. Wessells and Kostelny [18] recently coined the term Violence In Childhood (VIC), that also refers to how children who witness violence are impacted [19]. VIC may occur in home, school, community, social and cultural settings. 

The impact of prolonged political violence may be devastating to children’s well-being and development; however, its continued appearance may prompt civilians under fire and the community at large to be proactive; develop solutions for coping with the challenges; and provide children, families, and educational institutions suitable preventive resources, therapeutic interventions, and resiliency building routines [20] to support health and recovery, and bolster children’s sense of self-agency. 

The sense of potency among children living in environments of political violence 

Self-agency is the perception that the individual’s actions have an impact and influence the physical and relational environment [21]. Research shows that self-agency is shaped by the repeated behavior of the caregiver toward the infant or child (e.g., [22]). Specifically, when the infant experiences sensitive and good-enough parenting, he or she internalizes a sense of self agency, by which non-verbal cues bring positive changes (for example, a cry of hunger, will result in feeding). Two concepts related to self-agency, the first is self-efficacy [23], that describes the belief in one’s ability to fulfill tasks and goals, and the second is the concept of self-locus of control [24], which is the assumption that one can change reality. Self-efficacy was found as associated with motivation and cognitive achievements (e.g., [25,26]), as well as with social relationship aspects (e.g., [26,27]); In accordance with Ben-Sira [28], self-potency includes the dimensions of self-efficacy, and self-locus of control, and, in addition, one’s understanding that one’s social surroundings can be an important and significant anchor. To summarize, the sense of self-potency indicates the individuals’ sense of competency, based on various abilities and potential support from others. Self-potency may thus be regarded as a protective element that can support children’s successful adaptation and handling of aversive conditions, such as living in war zones. The concept is important from the aspects of health and self-resourcefulness; nevertheless, very few systematic study of self-potency has been undertaken [29], particularly in the context of war zones. Amongst the challenges of studying children’s self-potency is their limited ability to express themselves verbally.

### 1.3. Children’s Drawings as Communicating Their Experiences

Since most of the research-tools for assessing preschoolers’ emotional state and subjective experiences are verbal ones, and raters are mainly stakeholders such as parents and teachers, the present body of research in this field lacks the child’s perspective and voice [30]. The child’s perspective can be included by incorporating children’s non-verbal expressions, such as drawings. Drawings are a natural activity for children and engages them in exploration, communication, fun, and learning [31]. From a realistic perspective, children’s drawings progress gradually from scribbling to schematic and realistic images [32]. 

The current research included the drawings of children ages 4–6.9 years, a period of life which incorporates two developmental stages in drawing according to Lowenfeld and Brittain [33]. The first is the Preschematic stage, characterized by a conscious and clearly recognizable creation of form and objects, The child continually searches for new concepts and engages in problem solving, so symbols constantly change. The second developmental stage is the schematic stage, which characterizes the drawings of five- to six-year-old children, who draw schemas of specific objects in a repeated form, add a baseline [34], and emphasis objects by enlarging them. They also consider the space, demonstrated through variations in distance and how objects are organized [31]. Since the present study focuses on self-potency and emotional and cognitive aspects, the drawing task of a “Person Picking an Apple from a Tree” (PPAT: Gantt, [35]) was chosen, as it invites the child to depict a person that is in the process of reaching a goal. This drawing was found in previous studies to be related with children’s motivation, and executive functions [36], and with emotional and relational aspects [37]. For example, preschoolers’ executive functions problems were found to be related with a drawn person that was less active, while girls’ self-potency was associated with a successful apple picking [38]. The PPAT has reliable rating systems for form, content, and narrative aspects, and thus it can be incorporated in quantitative research format. The present study’s aim was to broaden our knowledge about children who live in areas of prolonged political violence by examining associations between children’s variables (cognitive and emotional) and maternal stress and their PPAT drawings (form, content, and narrative layers). The inclusion of the children’s drawings in this study incorporates their voices while enabling a closer inquiry into the explicit and implicit aspects of their subjective experience in relation to other data that was collected from their caregivers. This data triangulation may also strengthen and enrich the study’s results, and further deepen our understanding of how preschoolers communicate via their drawings subjective experience, strengths and difficulties that may need to be addressed. 

### 1.4. Research Hypotheses

Hypotheses of background variables:

1. Significant negative correlations were expected to be found between children’s self-potency, and emotional symptoms; executive functions (EFs) difficulties; and maternal stress.
2. Significant correlations were expected to be found between mothers’ assessments as to degree of exposure of their children to events of political violence, maternal stress, and child’s emotional symptoms.

Hypotheses regarding the child’s PPAT drawings

Since the child’s gender was found to be an intervening variable in previous studies, all the following hypotheses were also examined in relation to gender.

1. Significant positive correlations were expected to be found between child’s self-potency and positive content and form aspects in the PPAT.
2. Significant negative correlations were expected to be found between child’s EFs difficulties/ emotional symptoms and content and form aspects in the PPAT.
3. Significant correlations were expected to be found between maternal stress and negative aspects in the PPAT.
4. Significant correlations were expected to be found between the mothers’ assessments of the children’s degree of exposure to events of political and negative aspects in the PPAT.
5. The PPAT drawing’s narrative focus was expected to be less on the picking script in comparison to a sample of preschoolers in the same age group who live in a non-war zone [39]. In addition, the aspect of narrative focus was examined in relation to the child’s variables.

## 2. Method

### 2.1. Participants

156 preschool children (the age range of 122 children was: 4–6.9 years; Mean 5.32; SD = 0.75) who were recruited from randomly selected Israeli kindergartens located in an area affected by chronic political violence. Table 1 presents participants’ demographic variables. 

Table 1 about here.

As described in Table 1, 107 mothers (96% married; about 80% with an academic degree and 20% with a high-school education or diploma; 41% reported an average economic status; 34% an average plus economic status, and 19% a high economic status), and 15 kindergarten teachers participated in the study. This sample was recruited from an area near the Gaza strip, affected by political violence, mainly in the form of rocket attacks since the year 2001. Participants were mainly from small villages and kibbutzim (81%), while the minority were from the city of Sderot. The gender distribution of the children was equal. 

### 2.2. Instruments

#### 2.2.1. Mother’s Questionnaires

##### SDQ: Strengths and Difficulties Questionnaire [40]—Parent Version

A brief behavioral screening questionnaire assessing children’s (age 3–16 years) mental health, and social functioning. The questionnaire was translated to about 40 languages. The 25 items measure 5 scales: emotional symptoms, e.g., “Many fears, easily scared;” behavioral problems, e.g., “Often lies, and cheats;” hyperactivity/inattention, e.g., “Restless, overactive, cannot stay still for long;” peer relationship problems, e.g., “Rather solitary, tends to play alone;” and prosocial behaviors, e.g., “Considerate of other people’s feelings.” The items are coded on a three point scale: 0 = Not true, 1—Somewhat true, and 2 = Certainly true. The item’s score is calculated according to the online SDQ manual. In an extensive study of the psychometric characteristics of this tool, the measure was validated with DSM-IV diagnoses, and the five subscales were found as having a sufficient internal consistency (of 0.73) [41]. In the present study, only two scales had adequate internal consistency: the hyperactivity scale (Cronbach = 0.82), and the emotional problem scale, after omitting item no. 3 (Cronbach = 0.76). The internal consistency for the 19 items (omitting item no. 3, and the prosocial scale) was sufficient (Cronbach = 0.77). Scores were computed for each of the two scales and a total measure was computed, without the prosocial scale, and item no. 3.

##### BSI-18: Brief Symptom Inventory-18 [42]

An 18-item self-report questionnaire on psychological distress that was experienced in the previous week. The items are rated on a 5-point Likert scale ranging from 0 (not at all) to 4 (extremely). In the present study we used Canetti, et al. [43] in its Hebrew translation. The items covering three dimensions are measured through the subscales: somatization (e.g., “Pains in heart/chest”); depression (e.g., “Lonely”); and anxiety (e.g., “Scared for no reason”). Additionally, a Global Severity Index (GSI) score is also calculated [44]. Prior studies confirm that the BSI-18 has good internal consistency, as reflected in a Cronbach’s alpha of 0.89 [44,45]. There is adequate internal consistency for each of the scales: anxiety (0.71–0.79); depression (0.84–0.88); and somatization (0.74–0.80) [44,45]. The scales have adequate convergent and discriminant validity, constructing a three-factor structure (e.g., [44,46,47]). Adequate internal consistency was found in the current study for each scale: anxiety (Cronbach = 0.86); depression (Cronbach = 0.77); and somatization (Cronbach = 0.80). The internal consistency of the 18 items was high (Cronbach = 0.91). Scores were computed to each scale and for the total score.

##### The Child’s Exposure to Trauma [15]

The questionnaire included 7 items (yes/no answers) that refer to missile attacks and exposure to political violence (e.g., the child saw a missile fall, etc.), and two items that refer to nonpolitical trauma (e.g., the child was exposed to other dangerous/traumatic events). The sum of the total scores of political violence categories was used for further analysis.

#### 2.2.2. Teacher’s Questionnaires

Five Scales of the BRIEF Questionnaire [48], for Assessing EF Problems.

The children’s teachers rated 46 items relating to the child’s functioning during a time period of three weeks before and after the PPAT drawing, using a three—point scale (*N* = never, S = Sometimes, O = Often). The five scales that teachers evaluated included: initiating (e.g., “Needs to be told to begin a task even when willing.”); planning and organizing (e.g., “Has trouble concentrating on chores, schoolwork, etc.”); monitoring (e.g., “Has good ideas but cannot get them on paper.”); working memory (e.g., “When given three things to do, remembers only the first or last.”); and emotional control (e.g., “Erupts in rage for an insignificant reason”). The scales have high internal consistency (Cronbach = 0.90–0.93) and validity (*p* < 0.001) [48]. In the current study, the scales had high internal consistency (Cronbach = 0.88–0.95), and the global consistency was high as well (Cronbach = 0.98). A general score of the difficulties in executive functions was calculated as the total sum of these five scales. 

#### 2.2.3. Children’s Questionnaires

##### The CAMP: The Child Adaptation and Measure of Potency [49]

An interview-administered questionnaire, involves 19 potency statements which are answered orally by a yes or a no. The instrument’s statements were adapted from the adult’s Potency Scale [28], and were modified for children, by adding an opening question: “Which child resembles you?”. Each statement was formulated as positive and a negative, for instance: “A child who tries and succeeds” versus “A child who does not succeed even if he/she tries.” In cases when a child did not understand the statements, the researcher supplied examples for clarification. The measure has high internal consistency (Cronbach = 0.81), and a global score was computed. 

##### PPAT—Person Picking an Apple from a Tree Drawing [35]

Children were provided with white sheets of paper (21 cm × 29.5 cm) and 12 scented Sanford Mr. Sketch colors: red, orange, blue, turquoise, green, dark green, hot pink, magenta, purple, brown, yellow, and black. Each child was asked, separately, to draw a person picking an apple from a tree; no time limitation was set for the assignment. The PPAT drawings were scored based on three rating systems:

##### PPAT Drawings Analyses

*FEATS*—*Formal Elements Art Therapy Scale* [50]. This rating system is designed to measure the drawing’s form features. It uses twelve 5-point Likert scales: Prominence of Color; Color Fit; Implied Energy; Space; Integration; Logic; Realism; Problem-Solving; Developmental Stage; Details of Objects and Environment; Line Quality; and Person. A low score (0) on the Space scale represents a non-existent phenomenon, e.g., the absence of any drawing. A high score (5) reflects the prominence of this phenomenon, e.g., 100% of the space was used. 

Two art therapy graduate students underwent training in which they learned to rate PPAT drawings based on the FEATS Rating Manual [50]; Each of the students individually rated 40 PPAT drawings (which were not part of this study), and they reached a high inter-rater reliability calculated by ICC scores ranging between 0.72 to 0.95.

*SC-PPAT/c*—*Symbolic Content in PPAT drawings of children* [51]. The SC-PPAT/c comprises of 9 Likert scales for ranking the content-related characteristics of the tree, the person, and the relationship between them. The scales measure the quantity of apples on the tree; a tree’s strength vs. its weakness; the degree to which the person is active or passive in the apple-picking; the degree of success in picking the apple; amount of contact between the person and the tree; the height ratio between the person and the tree; the position of the tree trunk in relation to the person; the proximity of the branches to the person; and the proximity of the tree’s apples to the person. The Likert scales each have five to six points, depending on the scale. A low score (0) reflects a non-existent phenomenon, e.g., one of the objects is missing, while a high score (5 or 6) reflects the strong prominence of this phenomenon, e.g., a person with an apple in hand, which demonstrates success in the picking task. A previous study [38] revealed high inter-rater agreement and Interclass Correlations Coefficients (ICC) ranged from 0.78 to 0.96. In the current study ICC scores ranged between 0.84 to 0.99 (*n* = 35). After reaching inter-rater agreement, the rest of the drawings were divided between them for scoring.

*The Narrative Focus Degree Scale* [52]. A 6-point Likert scale measures the narrative focus of PPAT drawings. Narrative focus varies and ranges from drawings focusing only on the picking theme to drawings that contain a competing narrative that steers attention away from the picking theme. Drawings featuring the PPAT as the main theme receives a score of 1 to 4 that reflect an increasing PPAT narrative emphasis and richness. A score of (5) reflects the presence of a competing narrative, and a score of (0) reflects the absence of a picking narrative. Two art therapy students who were asked to rank 35 drawings reached high inter-rater reliability (ICC = 0.84).

### 2.3. Procedure

The Israeli Ministry of Education and the ethical committee of the University of Haifa’s Faculty of Social Welfare and Health Sciences approved the study. First, kindergarten teachers were invited to take part in this research, and then the children’s parents were contacted. Data was collected during 2018 and 2019. PPAT drawings [35], and the CAMP [49], were administered individually in the presence of a researcher. The children’s mothers completed the SDQ [40], which measures the child’s strengths and difficulties, the BSI-18 [44], which measures maternal stress, and a measure that assesses the degree to which their children were exposed to events of political violence [15]. Finally, the children’s teachers responded to five scales of the BRIEF questionnaire [48] that measures children’s problems in executive functions. 

## 3. Results

### 3.1. Preliminary Analyses and Descriptive Statistics—PPAT Drawings

Table 2 about here.

Table 3 about here.

**Figure 1:**
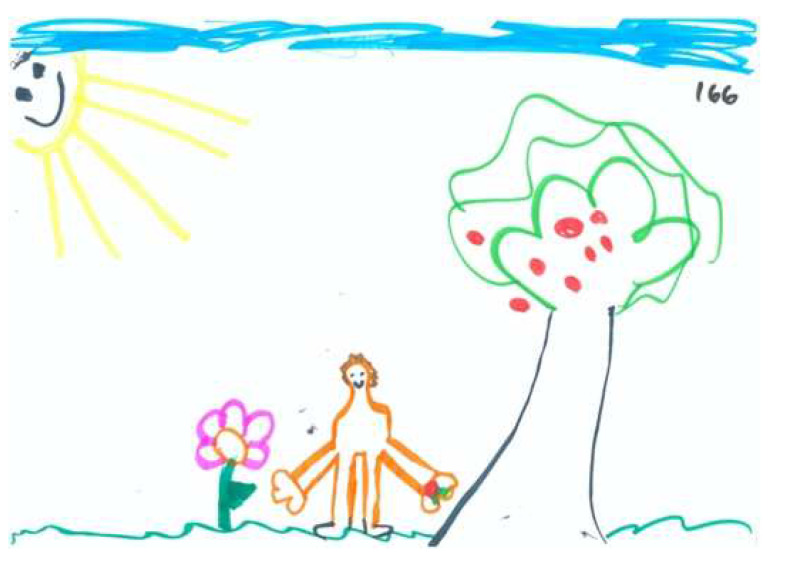
An average PPAT drawing, in regard to form and content layers.

Based on Table 2 and Table 3, the average drawing in this sample (see for example Figure 1) demonstrates the use of realistic color, with some of the drawn forms colored in. Children invested moderate energy in their drawings and used approximately 50% of the space. Most of the drawing reflect a development stage approaching the latency period; the items in the drawings were recognizable but simply drawn. The drawings contained only one unrecognizable item. The drawn person is holding an apple, but it is not clear how he/she obtained it. In addition to the person, tree, and apple, a horizon line/ground is perceptible, line quality is good (a controlled line), and there is integration of two to three elements. The drawn person is identifiable.

Observation of the content layer of the PPAT drawings reveals a fairly weak tree with 3 or 4 apples on it. The drawn person seems passive or is avoiding the picking process, and though the apple and person are close, there is no contact between them. The person is shorter than the tree by a ratio of 1:3. The tree truck is slightly inclined away from the person. Tree’s branches or treetop are neutral in relation to the person; however, they are high and inaccessible to the picker. The apples are placed on the side farther away from the person or distributed equally. 

Confirmatory Factor Analysis (CFA) was conducted using AMOS software version 23 for the FEATS and for the SC-PPAT scoring systems that compares the theoretical model and the empirical model (leaning on previous data, [39]). 

Table 4 about here.

Table 5 about here.

**Figure 2:**
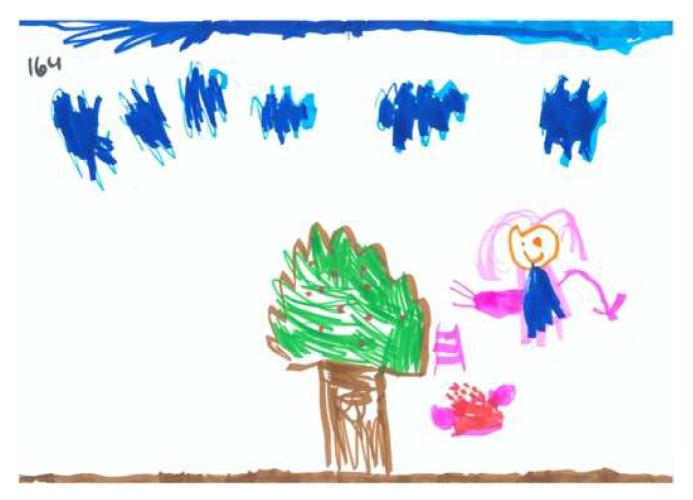
PPAT drawing with a high Vividness factor.

**Figure 3:**
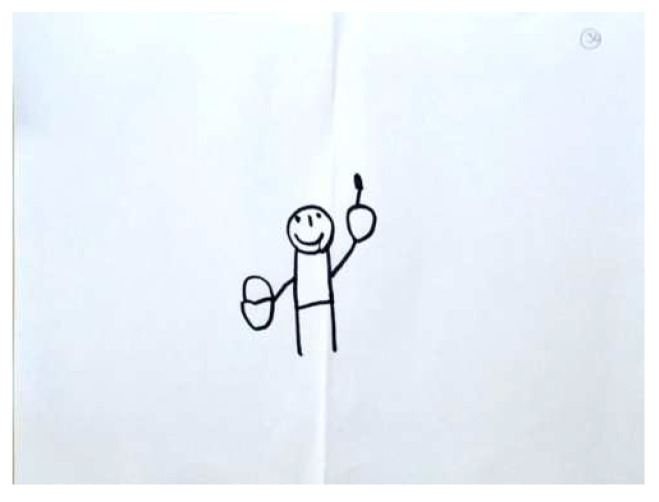
PPAT drawing with a low Vividness factor.

Regarding the FEATS scores, the CFA yielded acceptable goodness of fit indices. The first factor, Vividness, was identical in scale composition to this factor in previous sample [39], incorporating the following scales: Prominence of Color; Implied Energy; Space; and Details of Objects and Environment (Cronbach’s Alpha was 0.80). Vividness accounted for 40% of the common variance. Figure 2 and Figure 3 illustrate high and low levels of this factor respectively. 

**Figure 4:**
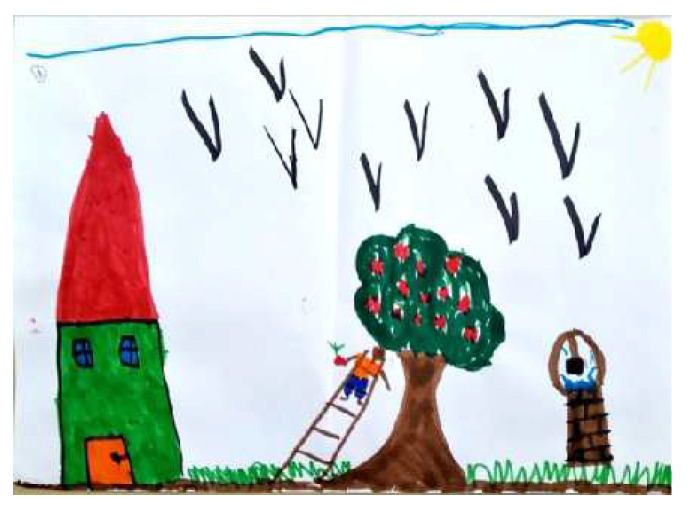
PPAT drawing with a high As-Real-R factor.

**Figure 5:**
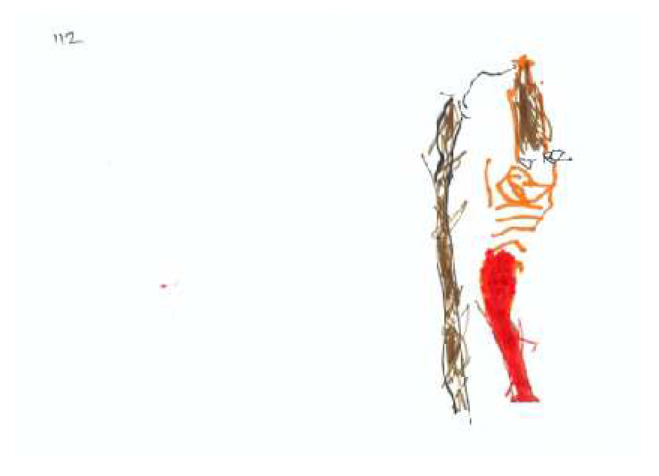
PPAT drawing with a low As-Real-R factor.

The second factor was found to incorporate more scales than the previous model; specifically, it included: Color Fit, Integration, Realism, Problem Solving, Developmental Level, Line Quality, and Person, with a good reliability (Cronbach’s Alpha was 0.73). This factor was named As-Real-R, because most of its scales serve the purpose of drawing recognizable images through the use of realistic colors, the relationships between the drawn objects, and a controlled line quality. R stands for Revised and different this factor from the original ‘As-Real’ factor in the study of Roth et al. [39]. Figure 4 and Figure 5 demonstrate high and low levels of this factor respectively. 

**Figure 6:**
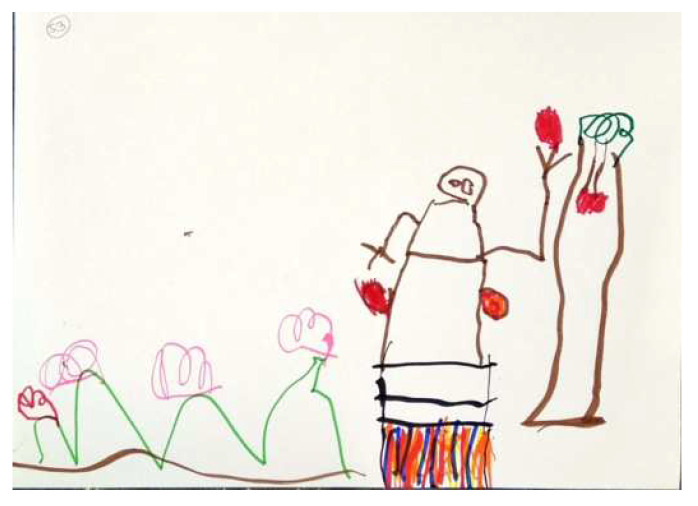
PPAT drawing with a high Person Agency factor.

**Figure 7:**
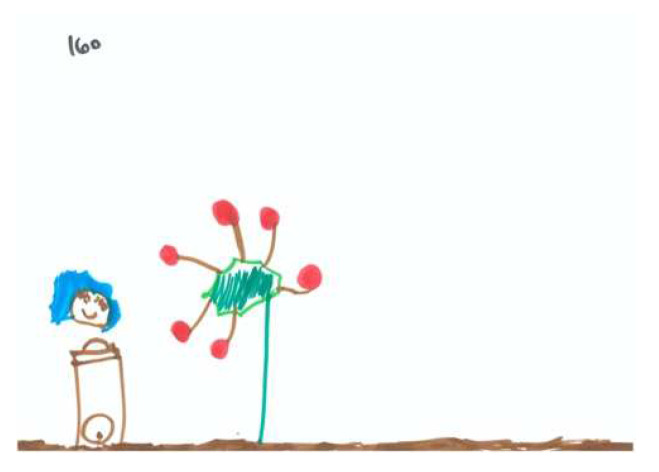
PPAT drawing with a low Person Agency factor.

Regarding the CFA of the SC-PPAT scores, we found no difference between the two models. As shown in Table 5, two main factors were obtained: ‘Person’s Agency’ describes the degree a person is active/passive in the apple picking process, and the degree of apple-picking success. Figure 6 and Figure 7 demonstrate high and low levels of this factor, respectively. 

**Figure 8:**
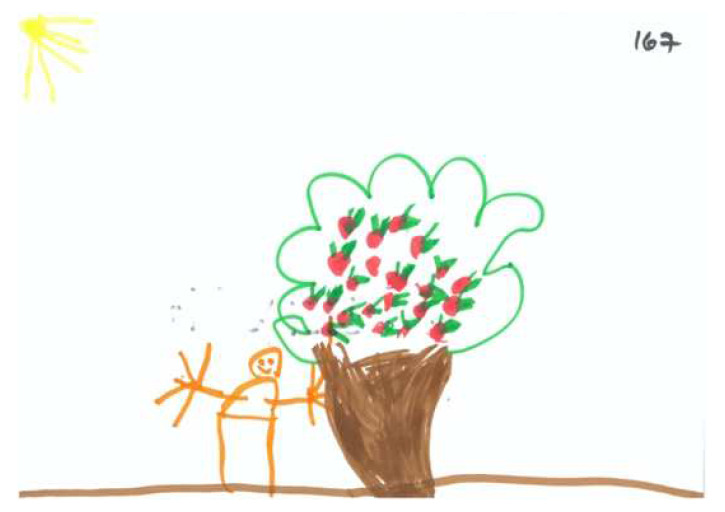
PPAT drawing with a high Tree Generosity factor.

**Figure 9:**
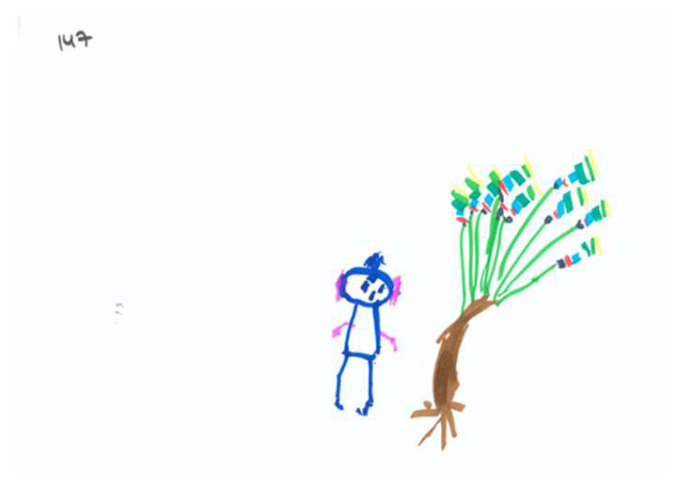
PPAT drawing with a low Tree Generosity factor.

‘Tree Generosity’ pertains to the tree’s strength, its number of apples, and the tree’s orientation is relation to the drawn person, including the trunk’s inclination, and placement of branches. Figure 8 and Figure 9 demonstrate high and low levels of this factor, respectively. 

These factors yield a total of 84% of the explained variance. 

**Figure 10:**
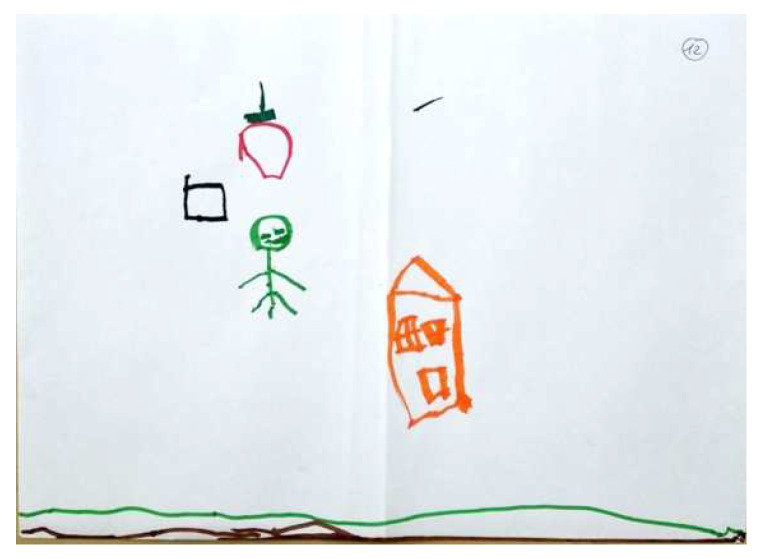
PPAT drawing with a missing apple-picking narrative.

**Figure 11:**
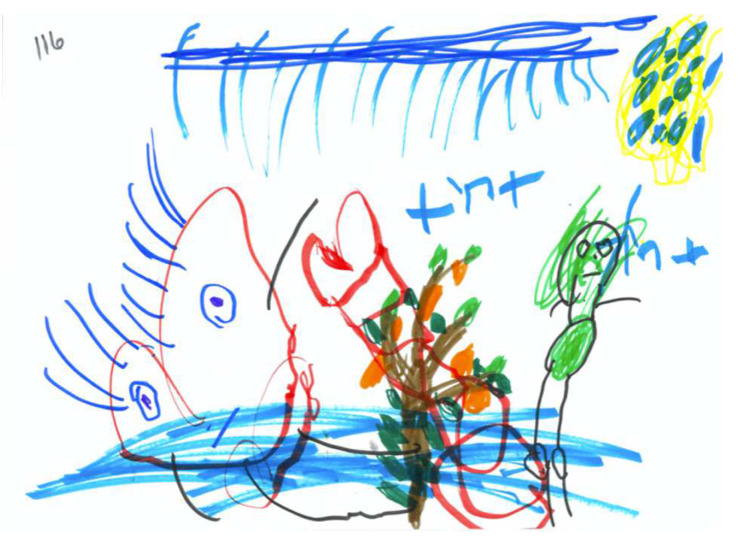
PPAT with a rival narrative.

Table 6 shows frequency score distribution of the PPAT drawing’s narrative focus. As can be seen the PPAT narrative was observed to be the main narrative in only 43.6% of the drawings. In about 28% of the PPAT drawings, the PPAT script was absent due to lack of at least one of the objects (a tree, a person, and/or an apple) (see for example Figure 10); in about 33% of the drawings, the children depicted narratives that rival the picking script and that became the central narrative (see for example Figure 11). The Mean score in this scale was 2.48 (SD = 2.03), i.e., on average the drawing showed a PPAT script that is limited to the picking script, with some degree of emphasis on one element of the script.

### 3.2. Preliminary Analyses and Descriptive Statistics—Independent Variables

Pearson correlations were calculated between the child’s variables with the goal of analyzing theoretical validity (Table 7), in specific, between the BSI global score of maternal stress, the SDQ sum of two scales that were found with high reliability (emotional symptoms, and hyperactivity), the BREIF total score summing the five scales, and the CAMP score of child’s self-potency. The results in Table 7 show that a significant association was found between the emotional problems of the child reported by the mother and EF problems reported by the child’s teacher (r = 0.438, *p* < 0.0001). Additionally, a medium significant association was found between the degree of maternal stress and the child’s emotional symptoms reported by the mother (r = 0.273, *p* < 0.004). Non-significant associations were found between the child’s self-potency, maternal stress, and child’s emotional symptoms. We also calculated these correlations in each gender group and found two additional associations only among girls: a positive association between maternal stress degree and girls’ EF problems (r = 0.351, *p* < 0.011), n = 52; and a negative correlation between girls’ EF problems and self-potency (r = −0.27, *p* < 0.036), n = 60. These associations partly confirmed the first hypothesis.

Spearman correlations were calculated between maternal assessments of the amount of political violence events that children witnessed and maternal stress and the child’s emotional and cognitive problems. As Table 8 shows, the number of events of political violence to which the child was exposed according to the mother’s assessment was positively correlated with the emotional problems she reported about her child (r = 0.362, *p* < 0.0001) and with her level of maternal stress (r = 0.309, *p* < 0.002). The last result confirms the second hypothesis, and indicates that the more the mother assessed that her child was exposed to events of political violence, the more she experienced maternal stress. 

### 3.3. Hypotheses Analyses Regarding the PPAT Drawings and Independent Variables

To examine hypotheses no. 3–5, we computed Pearson correlations between the child’s variables and the main factors of the drawings. Table 9 presents these associations.

Table 9 about here.

**Table 9:** Associations between the PPAT drawings’ main factors, and global scores of maternal stress and child’s variables, for the whole sample and for gender groups.

		Tree Generosity		Person Agency		Vividness		As-Real-R	
**Self-Potency (CAMP)**	Pearson Correlation	0.381 **		0.310 **		0.124		0.360 **	
	Sig. (2-tailed)	0.000		0.000		0.126		0.000	
	N	154		154		154		154	
	By Gender Girls|Boys	0.54 **	0.295 *	0.408 **	0.206	0.249 *	−0.075	0.560 **	0.178
	Sig. (2-tailed)	0.000	0.013	0.000	0.084	0.033	0.535	0.000	0.138
	N	74	71	74	71	74	71	74	71
**Emotional Problems (Total of two SDQ scales)**	Pearson Correlation	−0.214 *		−0.170		−0.134		−0.222 *	
	Sig. (2-tailed)	0.024		0.075		0.161		0.019	
	N	111		111		111		111	
	By Gender Girls|Boys	−0.081	−0.351 **	0.000	−0.283 **	−0.139	−0.055	−0.090	−0.241
	Sig. (2-tailed)	0.564	0.007	0.998	0.031	0.322	0.683	0.523	0.068
	N	53	58	53	58	53	58	53	58
**Difficulties in EFs (Total of BREIF 5 scales)**	Pearson Correlation	−0.421 **		−0.301 **		−0.192 *		−0.453 **	
	Sig. (2-tailed)	0.000		0.001		0.035		0.000	
	N	120		120		120		120	
	By Gender Girls|Boys	−0.274 *	−0.561 **	−0.124	−0.424 **	−0.108	−0.174	−0.176	−0.563 **
	Sig. (2-tailed)	0.034	0.000	0.347	0.001	0.413	0.186	0.180	0.000
	N	60	59	60	59	60	59	60	59
**Maternal Stress (BSI)**	Pearson Correlation	0.019		0.067		−0.077		−0.064	
	Sig. (2-tailed)	0.841		0.487		0.425		0.510	
	N	109		109		109		109	
	By Gender Girls|Boys	−0.119	0.247	0.054	0.071	−0.098	−0.105	−0.124	−0.044
	Sig. (2-tailed)	0.395	0.067	0.702	0.604	0.484	0.440	0.377	0.746
	N	53	56	53	56	53	56	53	56

** Correlation is significant at the 0.01 level (2-tailed). * Correlation is significant at the 0.05 level (2-tailed).

Hypothesis no. 3: As can be seen from Table 9, positive associations were found between the child’s self-report of self-potency, and three of the main aspects of PPAT drawings. In specific, the more the child saw herself/himself as self-potent, the more the PPAT drawing depicted a more generous tree (r = 0.38, *p* < 0.0001), a more active drawn person (r = 0.30, *p* < 0.0001), and the drawing was drawn in a more realistic manner (r = 0.36, *p* < 0.0001). These results confirmed the third hypothesis. Two of the associations were significantly stronger among girls than boys, according to Fisher Z of the difference between the correlation coefficients: specifically, the more a girl reported higher self-potency, the more her drawing was higher in As-Real-R factor (r = 0.56, *p* < 0.0001) z = 2.67 (*p* < 0.007), and higher in Vividness (r = 0.24, *p* < 0.033) z = 1.94 (*p* < 0.05). 

Hypothesis no. 4: Regarding children’s EF problems, negative associations were found between the child’s EF difficulties and the four main aspects of the PPAT drawing for the whole sample (associations range between −0.19 to −0.45). However, after a division according to gender, one significantly stronger association was found among boys (n = 59). Specifically, the more the teacher reported that a boy had EF difficulties, the more the boy’s PPAT drawing was lower in As-Real-R factor (r = −0.56, *p* < 0.0001) z = 2.44 (*p* < 0.01). 

In relation to the children’s emotional problems, small negative associations were found between the total score of emotional problems and two PPAT aspects (tree generosity and the drawing’s As-Real-R factor) for the whole sample. The correlations in each gender group were not significantly different according to Fisher Z. These associations (between EF and emotional problems and the PPAT drawings’ main factors) partly confirmed the fourth hypothesis.

Hypothesis no. 5: As Table 9 shows, we found non-significant associations between maternal stress and the child’s PPAT drawings, for the whole sample and for gender groups. For further examination, we also computed Pearson correlations for each sub-scale of the maternal stress measure, and we found a single positive association between the degree of maternal depression and the generosity of the boy’s tree (r = 0.28 *, *p* < 0.035). This means that the more the mother was depressed, the more her son drew a generous tree. This result showed that the fifth hypothesis was not confirmed.

Hypothesis no. 6: To examine the possibility of an association between the assessments of mothers regarding the degree of exposure of their children to events of political violence and PPAT drawings, we calculated Spearman correlations, and found that only among girls (n = 50), the higher the number of events of political violence that the daughter was exposed to according to her mother’s assessment, the more the daughter tended to draw an active drawn person in her PPAT drawing (r = 0.49 **, *p* < 0.0001), a generous tree (r = 0.28 *, *p* < 0.048), and a realistic drawing (r = 0.33 *, *p* < 0.016). The sixth hypothesis was disapproved. 

Hierarchical linear regressions were carried out to examine the impact of children’s gender, self-potency, and EF difficulties on the main factors of the children’s PPAT drawings. Gender was entered in the first block; in the second, self-potency and EF difficulties were entered using the stepwise method. Two statistically significant models were found as explaining the drawing’s As-Real-R factor, and the Tree Generosity (see Table 10 and Table 11). The results of the hierarchical linear regression analysis revealed that in regard to the Tree Generosity factor, gender was not a statistically significant predictor (*p* > 0.05); however, in regard to the As-Real-R factor, gender was found to be a statistically significant predictor.

Table 10 and Table 11 show the influence of gender, self-potency and EF difficulties on the degree of the drawing’s As-Real-R factor (F(3,115) = 18.55, *p* < 0.001) and the influence of self-potency and EF difficulties on the degree of Tree Generosity (F(3,115) = 19.27, *p* < 0.001). 

Specifically, the R2 indicated that 33% of the variance in the drawing’s As-Real-R degrees can be explained by variances in the three predictor variables: gender, self-potency and EF problems. For Tree Generosity, the R2 indicated that 34% of the variance in the Tree Generosity degree is explained by the child’s self-potency and EF difficulties. 

Hypothesis no. 7. As can be seen in Table 6, in drawings of children from the same age group from a non-war zone (n = 125) [39], the main narrative in the majority of the drawings (66%) was that of a person picking an apple from a tree (the percentage-sum of categories number 1 to 4); however, the current study revealed different results. Specifically, only 43.6% of the drawings depicted a central narrative of a person picking an apple. To calculate the difference of comparison of the proportions (of the two categories of missing picking narrative and rival narrative), a significant Chi-Squared of 14,084 was found *p* < 0.002.

The Focus of the Narrative scale was recoded into three main categories: (a) drawings lacking a picking scene (category 0; n = 35); (b) drawings depicting a picking scene as a the main narrative (categories 1–4; n = 68); and (c) drawings that depict a rival narrative as the central narrative (category no. 5; n = 51). We first conducted Spearman correlations between the three narrative categories and the child’s variables, including maternal stress, and then conducted further One-Way ANOVA tests (see Table 12), which revealed significant differences between the three narrative focus groups in terms of their self-potency scores F (2,115) = 12.05, *p* < 0.0001, Partial Eta Squared was 0.163. (Scores of self-potency for each narrative groups: Group 1. Missing picking narrative-Mean: 13.67, SD = 3.5; Group 2. A focused picking narrative-Mean: 16.60, SD = 2.24; Group 3. A rival narrative-Mean: 16.41, SD = 2.74). A post hoc Bonferroni test showed significant differences between Group 1 and the other two groups, i.e., children that drew a PPAT drawing without a picking narrative had the lower self-potency scores in comparison to children who drew a picking scene as the main narrative or a picking scene with a rival narrative.

Table 12 about here.

**Table 12:** Univariant ANOVA for self-potency differences withing the three narrative categories.

Tests of between-Subjects Effects						
Dependent Variable: Self Potency (CAMP)						
Source	Type III Sum of Squares	df	Mean Square	F	Sig.	Partial Eta Squared
Corrected Model	220.677 ^a^	2	110.339	14.723	0.000	0.163
Intercept	34,659.932	1	34,659.932	4624.984	0.000	0.968
PPAT Narrative Focus: 3 groups	220.677	2	110.339	14.723	0.000	0.163
Error	1131.604	151	7.494			
Total	40,154.750	154				
Corrected Total	1352.281	153				

^a^ R Squared = 0.163 (Adjusted R Squared = 0.152).

## 4. Discussion

The present study sought to investigate the experiences of preschool children living in areas of prolonged political violence through examination of their PPAT drawings in relation to various child and maternal variables.

Regarding the children’s mothers’ perspective, significant associations were found between mothers’ reports on the degree to which their children were exposed to events of political violence and the degree of maternal stress. According to caregiving theory, one of the parent’s roles is to keep the child safe for the purpose of her/his survival [53], as well as to serve as a secure haven, helping the child in times of need to cope with stress and anxiety [54]. However, within the context of political violence, the parent may feel that his/her ability to fulfill the two roles is compromised, and thus experience heightened emotional distress (e.g., [55]). This may trigger a cycle in which the maternal caregiver’s functions- for instance maternal responsiveness and sensitivity- are limited due to higher maternal stress, and this in turn may have a negative effect on the child’s well-being (e.g., [10,55]). Accordingly, the current study found that the more mothers reported experiencing stress, the more they tended to report that their child suffers from emotional problems. Although parent’s reports may be biased by their own distress and symptomatology [56], significant associations were found in the present study between the mother’s and teacher’s reports about children’s difficulties. Thus, the current study confirms the presence of associations between maternal stress and the child’s emotional problems. In regard to maternal stress and child’s variables, we found that among girls only, the more the mother felt stress, the more EF problems her daughter has, and the more girls had EF problems, the lower their sense of self-potency. Bearing in mind the small sample size of the current study, particularly in the context of the effects of gender, further research is required in order to determine whether girls’ executive functioning is vulnerable to the effects of maternal stress or distress. 

Regarding children’s self-potency, which was assessed from the child’s perspective in this study, we found a surprising finding of no association between maternal stress and the child’s self-potency. We would like to present three possible explanations for this finding. First, from a developmental perspective, the child’s sense of self-agency is formed early in her/his development, and this may show continuity despite contextual liabilities over time (e.g., [57]). Second, the present study did not measure additional parental aspects that are known to have an impact on the child’s variables in the context of conflict, for example parental support and monitoring [58,59]. In addition, the present study did not include paternal variables that have a crucial influence in the context of the triadic relationship, and in particularly during stressful events, when the child might calm down as a result of reaching out to the other parent, or even by watching her/his parents in a calm dialogue or discussion about the situation [60]. The third explanation leans on the ecological perspective which takes into account the potential of the community to mitigate the effects of armed conflict [61,62]. Specifically, in the present study, the children and their families live within mainly small collective communities that have already established social and community initiatives and services designed to help their members cope with the daily events of violence [63]. Thus, we can suppose that the community, which also includes members of the kindergarten staff, functions as a buffer and as a protective factor in regard to the children’s sense of self-potency. This speculation is in accordance with one of the dimensions of the self-potency concept- which includes the individual’s perception of the social envelope as a source of support in times of need [28].

### 4.1. The Drawings as a Source of Insight 

The current study sought to examine associations between PPAT drawings of normative and functioning children who live in an area of persistent political violence and children’s and maternal variables. The study’s results illuminate the potential clinical-assessment value of careful observation of positive and negative aspects of children’s PPAT drawings in relation to emotional strengths and cognitive difficulties. The drawings were analyzed and coded quantitatively into art formal elements, content aspects and categories of narrative focus.

Although the form and content aspects were similar to those found in a normative sample of the same age group that lives in a non-war zone in Israel [39], when we examined the focus of the drawings’ narrative, we found significant differences: the current sample had more drawings lacking a picking scene narrative, and more drawings with a rival narrative diverting the observer’s attention away from the apple-picking scene. These differences may be explained by the unique context that may have interfered and reduced the children’s focus on the task’s requirements. This could happen for example when children felt preoccupied with other issues, and/ or were overwhelmed with feelings of unsafety, or were vigilant or fearful in the context of living in an area of political violence. Bearing in mind that the drawing task was conducted in the kindergarten setting with an unfamiliar adult (the researcher), it could have been experienced by the children as a stressful event (e.g., [64,65]). Although the same procedure was used in the previous study [38], it may be speculated that the children in the conflict zone suffer from the cumulative effects of stress and this affected their performance. Psychoanalytically-speaking, war interferes with the continuous flow of everyday life and impairs one’s sense of personal safety; thus, it may impact attention and perception [66].

Sandler [67] has introduced the concept of ‘background of safety,’ as a feeling generated from our perceptions:

“the successful act of perception is an act of integration that is accompanied by a definite feeling of safety—a feeling so much a part of us that we take it for granted as a background to our everyday experience”.

(p. 353)

Gampel [66]), who researches the experiences of traumatized individuals, has identified the opposite experience, the ‘background of the uncanny’ which emerges from situations of living in contexts of social violence. Overwhelmed by horror and pain, our precepting and symbolization abilities are impaired. Neuroscience has shown that long-term and chronic stress are related to the heightened secretion of cortisol levels, which may negatively affect cognitive functions by changing processes of neurotransmission in the hippocampus and the prefrontal cortex [68]. We can thus speculate the abilities of 56% of the children who took part in the present study and who drew a missing picking scene and a rival narrative were compromised, and they could not respond accurately to the requirements of the task. Furthermore, it can be speculated that these children communicated authentic subjective experiences in their drawings, such as the experience that something is missing, and thus fragmented, as in the cases of the missing picking narrative; they also communicated the need to process other “burning” issues that they were coping with, such as by drawing PPAT drawings with a rival narrative. 

An additional important finding in this study was that children who drew PPAT drawings with a missing picking narrative reported the lowest self-potency in this sample. This could indicate that these children, whose PPAT drawings revealed a deficient triad (absence of a person, or tree, or apple), implicitly communicated the basic experience of being unable to reach a goal, or to be part of an effective triadic alliance. These are the children that might be in a need of therapeutic intervention to strengthen their sense of self-potency.

The present study also showed evidence of associations between PPAT drawings and emotional and cognitive aspects; in some cases, gender was an intervening variable. The child’s experience of self-potency was found to be positively associated with positive aspects in the PPAT drawings; specifically, the more the child perceived herself/himself as self-potent, the more the drawn tree was generous, the drawn person was active, and the drawing was realistic. In addition, the PPAT drawings tended to be more vivid among girls with stronger self-potency. The differences in the expressions of positive experiences of potency between the two genders may be related to meta-analysis reviews showing that girls express more positive emotions than boys (e.g., [69,70]). As mentioned above, in a previous study with the same age-group in Israel in a non war-zone [38]. we found associations between self-potency and positive content in the PPAT drawings only among girls. 

Children’s difficulties, as assessed by teachers and mothers, were inversely associated with the PPAT drawing’s main factors; specifically, as much as the child coped with cognitive and emotional problems, the less generous the drawn tree was, and the less realistic the drawing. EF difficulties were also found to be associated with a less active drawn person, and a less vivid drawing. The present study used a regression model for calculating the explained variance of the PPAT drawings by cognitive and emotional child’s variables. Findings show that the children’s cognitive difficulties and their self-potency experiences explain only about 30% of the variance of the As-Real-R and the Tree Generosity factors. This indicates that there are additional aspects—not measured in the current study—that may explain the variances of these drawing, for example, creativeness; motivation; cognitive abilities; social abilities; and environmental variables, such as art, education, and more.

In summary, these results may be described as a parallel-track visual communication pattern, in which children express their personal strengths through positive form and content aspects of the drawing, and express difficulties through negative form, content and narrative aspects of the drawing. 

This pattern was found in many studies that showed that positive visual drawing elements were associated with child’s strengths and abilities, and negative visual drawing element were associated with children’s emotional difficulties (e.g., [71,72,73]). Evidence of this parallel-track visual communication was also found in studies of children’s PPAT drawings, for example, poor line quality and less color prominence were associated with children with ADHD [74]; a less active drawn person was associated with EF difficulties, while an active drawn person was associated with higher self-potency in preschoolers [38] and with maternal hostility/aggression among school-age children [37].

The present study, however, also identified an inverse pattern, where negative child/maternal variables were associated with positive expressions in the PPAT drawings. These may be understood as hope expressions, correcting or compensating efforts, and/or wish fulfillments representations in the face of experiences of adversity and helplessness. In this pattern, we found gender to be an intervening variable. Specifically, the more the mother reported feeling depressed, the more her son tended to draw a more generous tree, and the higher the exposure of the daughter to events of political violence according to the mother, the more the daughter drew a more active person, a more generous tree, and a more realistic drawing. Psychoanalytic theory perceives compensating expressions/behaviors—for example, the phenomenon of an imaginary friend that functions as a buffer against loneliness—as non-adaptive; moreover, when the child relates to the imaginary friend in a concrete way, it also prevents him/her from establishing real relationships [75]. In the same vein, artistic products are interpreted as expressing contents of the unconsciousness as wishes and expressions of the forbidden (e.g., [76]). Furthermore, children’s drawings may demonstrate children’s defense mechanisms [77]. However, drawing could serve as a space in which the child could engage in intellectual play [78], and/or immerse himself or herself in the potential space and move along the bridge that connects reality and phantasy [79]. Accordingly, the child could feel he or she is a problem solver, a creator and one who controls the drawing medium [80]. Symbolic expressions of hope and overcoming may be understood as child’s attempts to self-soothe, regulate emotions, have fun and make the shift from helplessness to agency. Thus, further research is recommended to better understand the nature of the positive images and visual elements in the PPAT drawing in the face of negative life contexts; specifically, do they serve as non-adaptive compensations defenses, or expressions of hope and positive thinking that may be part of adaptive efforts.

The present study’s findings—that of a parallel-track communication pattern reflecting child’s difficulties and strengths through negative and positive aspects in their drawings versus compensating positive expressions despite experiences of deficit and stress—may demonstrate the complex nature of the individual’s drawing as it relates to multiple and sometimes contradictory phenomena. These findings may also reinforce McGrath & Carroll’s [81] tenet that projective tasks, including projective drawings, may serve as a portal for the broadband implicit representations of the individual, and not as valid personality tests. This implies that extra caution is needed when endeavoring to draw conclusions from children’s drawings.

### 4.2. Study’s Limitations, Future Recommendations, and Clinical Implications

The present study’s sample size was 156 children, however teachers and mothers’ compliance rates were low, so sample size was reduced when calculating some of the associations and regression tests. We thus recommend conducting this research with a larger sample size, and in particularly, to examine the gender differences that were found in the current study. Secondly, we did not measure paternal variables, and this may be a limitation due to the fact that both parents affect the way children function in many areas. Another limitation is that we did not measure child’s behavior factors in times of stressful events, for example behavior of comfort-seeking was found as associated with child’s better functioning in a war-zone [82]. This article, because of space limitations, does not include a description of the processes of drawings and the accompanying verbal stories of these children that were also documented. 

Regarding clinical implications, the present study makes three main contributions. The first is the ability of PPAT drawing to communicate issues of self-potency, particular drawings that lack the picking narrative. The second is the potential to gain insight about children by observing the narrative of their PPAT drawings. In cases of a missing picking narrative or the presence of a rival narrative, the clinician should ask herself/himself: What is going on? Why is the drawing incomplete? What is the meaning of the absence of an element? What is the meaning of the additional predominant narrative? Thus, the narrative focus may serve as a source of questions and associations for the clinician, as part of her/his effort to better understand the child. The third contribution is addressed towards the psychotherapist who works with children’s drawings. The findings which reveal the presence of a parallel-track communication pattern, along with compensating and/or hope expression pattern, demonstrate that each drawing may be the individual child’s idiosyncratic expression. This means that in our endeavor to understand the messages the child communicates through his/her drawing, we need to carefully observe each nuance in the drawing, whether it is in the form or content layers, and determine if it illustrates a positive aspect reflecting the child’s strengths or a compensating or hope expression in the face of adversity. During the therapeutic process, after the child draws the PPAT drawing, the clinician may ask questions or even invite the child in a playful manner to draw, for example: “What happened next?”; “what happened before?”. These questions/invitations may serve as therapeutic interventions in the case of a picking narrative that is lacking, or the depiction of a passive figure, and may thus enliven the narrative and encourage the child’s expressions of self-potency.

## 5. Conclusions

The present study shows that the PPAT drawings of preschool children who live in area of persistent political violence convey the children’s strengths and difficulties through layers of form, content, and narrative. The narrative focus’s analysis of these drawings revealed that these children express much more either competing narratives and either the absence of a picking script, than children who live in a non-war zone. These specific narratives may be interpreted as a call for further attention to the child’s self-potency and unique needs as they may arise in the context of the ‘background of the uncanny’ [66]. The associations between the PPAT drawings main factors and the child’s variables that were found in the present study reveal a complex communication dynamic. Specifically there are parallel patterns pertaining to the child’s strengths and difficulties as they were associated with positive and negative visual aspects in the drawings, respectively, as well as the presence of compensating expressions, reflected in the positive visual aspects created despite adverse context’s variables. These expressions may be interpreted as resisting responses against helplessness, and/or restoring actions towards safety, at least in the child’s creative space. Further research is required to illuminate on these two patterns; for example, what are the characteristics of children who lean more on parallel versus compensation patterns in their PPAT drawings? What is the role of gender in this respect? Is resiliency related to these forms of communication? Finally, if it is, can we use children’s drawings to learn more about how they are coping in the face of stressful event? And how can we further foster their coping skills through art-based interventions?

## Figures and Tables

**Table 1:** Demographic Data: Means, SDs (in Parentheses), and Percentages.

Variable, *N* and *N* Missing	Indices and Percentages
Child’s age (in years) *N* = 122 (34 missing)	Mean = 5.32 (SD = 0.75) Range 4–6.9 years
Gender of children (%) *N* = 147 (9 missing)	47.8% girls 46.8% boys
Socioeconomic status *N* = 105 (51 missing)	Mean = slightly above average level
Mothers who completed higher education (%) *N* = 107 (49 missing)	80%
Marital status (% married mothers) *N* = 107 (49 missing)	96%
Locality (% rural) *N* = 156	81%

**Table 2:** Ranges, Means, Standard Deviations of the FEATS scales (*N* = 156).

Scale No.	FEATS Scales	Min	Max	Mean	SD
1	Prominence of Color	1	4	2.28	1.10
2	Color Fit	0	5	4.09	1.09
3	Implied Energy	0	5	2.96	0.94
4	Space	0	5	3.16	1.13
5	Integration	0	4.5	3.02	1.03
6	Logic	0	5	4.12	1.24
7	Realism	0	4	2.64	0.79
8	Problem Solving	0	5	2.07	1.61
9	Developmental Level	0	4	2.52	0.49
10	Details of Objects & Environment	0	5	2.13	1.38
11	Line Quality	2	4.5	3.38	0.56
12	Person	0	5	3.40	1.43

**Table 3:** Means, Standard Deviations of the SC-PPAT/c: Symbolic Contents in children’s PPAT [51] (*N* = 156).

Scale No.	Measure	Points on Likert Scale	Score No. 1	Score No. 5 or 6	Mean	SD
1	Quantity of apples on the tree	6	A tree with no apples	Above ten apples on the tree	3.22	2.10
2	Tree’s strength vs. weakness	5	A very weak tree (more than 3 weakness indicators)	A very strong tree (more than 3 strength indicators)	2.29	1.62
3	The degree to which the person is active in apple-picking	5	The person clearly avoids picking (e.g., turned in another direction)	The person is clearly active in the picking process, plus stands on a heightening object	2.35	1.52
4	Degree of success in picking the apple	5	There is no closeness nor touch with the apple, or there are no apples on tree.	The person holds one or more apples, and/or the apple/s is/are in a basket/container	1.98	1.57
5	Contact between the person and the tree	4	There is one point of contact between the person and tree/apple	The person is actually within the contour of the tree	1.21	0.97
6	Height ratio between person and tree	6	The person is significantly shorter than the tree (1:5)	The person is taller than the tree (2:1)	3.25	2.02
7	Position of tree trunk in relation to the person.	5	The tree trunk is inclined away from the person.	The tree trunk is inclined toward the person.	2.22	1.39
8	Branch (or tree top) placement in relation to the person (close vs. far).	5	Branches placed on the side of the tree farther from the person.	Branches stem from the trunk, towards the person (clearly assisting).	2.30	1.60
9	The extent to which apples are scattered on the tree either close or far from the person	5	All apples are placed on the side farther from the person	All apples are placed on the side closer to the person	2.62	1.75

**Table 4:** Confirmatory Factor Analysis of FEATS scales.

Measure		Factor	Estimate
Prominence of Color	<---	Vividness	0.419 ***
Implied Energy	<---	Vividness	0.719 ***
Space	<---	Vividness	0.666 ***
Details of Objects & Environment	<---	Vividness	0.906 ***
Integration	<---	As-Real-R	0.699 ***
Realism	<---	As-Real-R	0.714 ***
Problem Solving	<---	As-Real-R	0.306 ***
Developmental Level	<---	As-Real-R	0.878 ***
Color Fit	<---	As-Real-R	0.553 ***
Person	<---	As-Real-R	0.452 ***
Line Quality	<---	As-Real-R	0.369 ***

χ^2^ (36) = 84.10, *p* < 0.0001, CFI = 0.936, RMSEA = 0.093, SRMR = 0.067. Correlations between the two latent variables r = 0.734. *** *p* < 0.001.

**Table 5:** Confirmatory Factor Analysis of SC-PPAT/c2 scales.

Measure		Factor	Estimate
The degree to which the person is active/passive in apple-picking	<---	Person’s Agency	0.985 ***
Degree of success in picking the apple	<---	Person’s Agency	0.862 ***
Quantity of apples on the tree	<---	Tree’s Generosity	0.543 ***
Strength vs. weakness of tree	<---	Tree’s Generosity	0.541 ***
Position of the tree trunk in relation to the person	<---	Tree’s Generosity	0.893 ***
Branch/s placement in relation to the person	<---	Tree’s Generosity	0.890 ***
The extent to which apples are scattered on the tree either close or far from the person	<---	Tree’s Generosity	0.836 ***

χ^2^ (13) = 22.85, *p* = 0.043, CFI = 0.98, RMSEA = 0.070, SRMR = 0.046. Correlations between the two latent variables r = 0.746. *** *p* < 0.001.

**Table 6:** Frequencies of Degree of Narrative Focus [52] *N* = 156 in comparison to the prevalence in percentages from a sample of the same age from a non-war zone [39] *N* = 125 in parenthesis. Adapted with permission from [39] 2020 Elsevier Ltd.

Score	Category	Description	Frequency	Valid Percent	Cumulative Percent
0	Absence of PPAT narrative message	Partial or complete absence of picking aspects	37	23.7 (12)	23.7
1	Schematic and balanced PPAT narrative message	The drawing focus solely on the PPAT story, with equal investment in all parts of the drawing	30	19.2 (20)	42.9
2	Emphasized narrative message	The drawer emphasizes certain details about the person/tree/ picking/apples/accessories/environment (in this example the trunk)	20	12.8 (28)	55.8
3	Narrative message supported by the environment	The environment supports the picking process sun, plants, flowers, etc.	9	5.8 (4)	61.5
4	Narrative message of PPAT reflected by other/s	The presence of a live audience watching the picking (e.g., a turtle.)	9	5.8 (12)	67.3
5	Competing/rival narrative message	The presence of elements that distract the attention of the apple picker (e.g., a snake approaching the picker)	51	32.7 (21)	100.0

**Table 7:** Associations between global scores of child’s problems in EFs, emotional symptoms, self-potency, and maternal stress, for the whole sample and for gender groups.

Correlations					
		Maternal Stress (BSI)	Emotional Problems (Total of Two SDQ Scales)	Difficulties in EFs (Total of BREIF 5 Scales)	Self-Potency (CAMP)
Maternal Stress (BSI)	Pearson Correlation	1	0.273 **	0.128	−0.007
	Sig. (2-tailed)		0.004	0.194	0.945
	N	109	109	105	109
Emotional Problems (Total of two SDQ scales)	Pearson Correlation	0.273 **	1	0.438 **	−0.001
	Sig. (2-tailed)	0.004		0.000	0.995
	N	109	111	107	111
Difficulties in EFs (Total of BREIF 5 scales)	Pearson Correlation	0.128	0.438 **	1	−0.121
	Sig. (2-tailed)	0.194	0.000		0.188
	N	105	107	120	120
Self-Potency (CAMP)	Pearson Correlation	−0.007	−0.001	−0.121	1
	Sig. (2-tailed)	0.945	0.995	0.188	
	N	109	111	120	154

** Correlation is significant at the 0.01 level (2-tailed).

**Table 8:** Associations between the mother’s assessment of political violence events, with global scores of maternal stress and child’s variables.

Correlations						
			Emotional Problems (Total of Two SDQ Scales)	Difficulties in EFs (Total of BREIF 5 Scales)	Maternal Stress (BSI)	Self-Potency (CAMP)
Spearman’s rho	Political violence events—amount assessment	Correlation Coefficient	0.362 **	−0.010	0.309 **	−0.033
		Sig. (2-tailed)	0.000	0.921	0.002	0.740
		N	102	99	100	102

** Correlation is significant at the 0.01 level (2-tailed).

**Table 10:** Multiple Linear Regression Stepwise (Dependent Variable: As-Real-R Factor in PPAT).

Variable	Model 1			Model 2			Model 3		
	B	SE B	β	B	SE B	β	B	SE B	β
Gender	0.34 **	0.12	0.25 **	0.22	0.14	0.16	0.20	0.11	0.15
Difficulties in EFs (BRIEF) ^b^				0.02	0.003	−0.42 ***	−0.01	0.003	−0.39 ***
Self-Potency (CAMP) ^c^							0.07	0.018	0.31 ***
F Change	7.68 **			25.71 ***			16.08 ***		
R^2^ (Adj. R^2^)	0.06 (0.05)			0.23 (0.22)			0.33 (0.31)		
R^2^ Change	0.06			0.17			0.09		

Gender 1 = Female. ^c^ CAMP Questionnaire; ^b^ BRIEF Questionnaire. ** *p* < 0.01; *** *p* < 0.001.

**Table 11:** Multiple Linear Regression Stepwise (Dependent Variable: Tree Generosity in PPAT).

Variable	Model 1			Model 2			Model 3		
	B	SE B	β	B	SE B	β	B	SE B	β
Gender	−0.15	0.25	−0.06	−0.40	0.23	−0.15	−0.45	0.21	−0.17 *
Difficulties in EFs (BRIEF) ^c^				−0.03	0.006	−0.45 ***	−0.03	0.006	−0.41 ***
Self-Potency (CAMP) ^d^							0.17	0.04	0.37 ***
F Change	0.36			28.24 ***			23.56 ***		
R^2^ (Adj. R^2^)	0.003 (−0.005)			0.20 (0.18)			**0.34 (0.32)**		
R^2^ Change	0.003			0.20			**0.14**		

Gender 1 = Female. ^d^ CAMP Questionnaire; ^c^ BRIEF Questionnaire. * *p* < 0.05; *** *p* < 0.001.
